# Enhancement of therapeutic efficacy of Brinzolamide for Glaucoma by nanocrystallization and tyloxapol addition

**DOI:** 10.1186/s40780-024-00375-5

**Published:** 2024-09-05

**Authors:** Shuya Masuda, Shiho Yano, Tomohisa Tadokoro, Hiroko Otake, Noriaki Nagai

**Affiliations:** 1 Faculty of Pharmacy, Kindai University, 3-4-1 Kowakae, Higashi-Osaka, 577-8502 Osaka Japan; 2 Pharmaceutical Research Laboratories, Senju Pharmaceutical Co., Ltd, 6-4-3, Minatojima-Minamimachi, Chuo-Ku, Kobe, 650-0047 Hyogo Japan

**Keywords:** Brinzolamide, Nanocrystal formulation, Ophthalmic drug delivery, Corneal permeability, Tyloxapol

## Abstract

**Background:**

Brinzolamide (BRI) suspensions are used for the treatment of glaucoma; however, sufficient drug delivery to the target tissue after eye drop administration is hampered by poor solubility. To address this issue, we focused on nanocrystal technology, which is expected to improve the bioavailability of poor-solubility drugs, and investigated the effect of BRI nanocrystal formulations on corneal permeability and intraocular pressure (IOP)-reducing effect.

**Methods:**

BRI nanocrystal formulations were prepared by the wet-milling method with beads and additives. The particle size was measured by NANOSIGHT LM10, and the morphology was determined using a scanning probe microscope (SPM-9700) and a scanning electron microscope (SEM). Corneal permeability was evaluated in vitro using a Franz diffusion cell with rat corneas and in vivo using rabbits, and the IOP-reducing effect was investigated using a rabbit hypertensive model.

**Results:**

The particle size range for prepared BRI nanocrystal formulation was from 50 to 300 nm and the mean particle size was 135 ± 4 nm. The morphology was crystalline, and the nanoparticles were uniformly dispersed. In the corneal permeability study, BRI nanocrystallization exhibited higher corneal permeability than non-milled formulations. This result may be attributed to the increased solubility of BRI by nanocrystallization and the induction of energy-dependent endocytosis by the attachment of BRI nanoparticles to the cell membrane. Furthermore, the addition of tyloxapol to BRI nanocrystal formulation further improved the intraocular penetration of BRI and showed a stronger IOP-reducing effect than the commercial product.

**Conclusions:**

The combination of BRI nanocrystallization and tyloxapol is expected to be highly effective in glaucoma treatment and a useful tool for new ophthalmic drug delivery.

## Background

Visual information accounts for more than 80% of the information provided to the brain, and a loss of visual function due to ocular disease leads to a marked reduction in the quality of life. The main treatment for ocular diseases is drug therapy. However, the eye has a blood-aqueous barrier and a blood-retinal barrier, which prevent the uptake of substances from the blood [1, 2]. Therefore, it is difficult to deliver drugs into the eye by systemic administration, and the primary route of administration for ocular disease treatment is topical administration by eye drops. However, there are also several known problems for the treatment of ocular diseases by eye drops. Drugs administered by eye drops are diluted by tear fluid and washed away from the ocular surface through the nasolacrimal duct [3]. In addition, the cornea has a strong barrier function that prevents foreign substances from entering the eye, and less than 1% of the drug administered by eye drops is delivered into the eye [4]. Therefore, there is a need for technology to efficiently deliver drugs into the eye by ophthalmic administration.

Nanotechnology is a general term for technology that controls nano-sized substances, and nanoparticles are known to improve the bioavailability (BA) of drugs [5]. In the field of ophthalmology, drug delivery systems (DDS) using nanotechnologies, such as nanocrystals, polymer nanoparticles, liposomes, niosomes, and dendrimers are actively being investigated [6]. Among them, the nanocrystals can efficiently deliver the required therapeutic dose because the drug itself is nano-sized rather than using nanoparticles as drug carriers. We have previously demonstrated that drugs with a controlled particle size of less than 200 nm have high corneal penetration and reported that endocytosis is involved in the enhancement of the corneal permeability of nanocrystals [7–9].

Glaucoma is an ocular disease that causes irreversible visual impairment, and high intraocular pressure (IOP) is strongly associated with the onset and progression of primary open-angle glaucoma (POAG) [9]. In POAG treatment, medical treatment with eye drops is considered the first line-treatment [10]. Brinzolamide (BRI), a carbonic anhydrase inhibitor (CAI), is frequently used as a treatment for glaucoma because it reduces IOP by decreasing aqueous humor production [11–13]. Meanwhile, the solubility of BRI is very low (0.4 mg/mL, pH 7.4) [14], so the commercial formulation, Azopt®, is a micro-sized (> 10 μm) eye suspension. Therefore, compared to aqueous eye drops, it is more susceptible to clearance on the ocular surface and has lower corneal permeability. Thus, BRI is one of the most desired ophthalmic agents for glaucoma treatment to improve corneal permeability. As mentioned above, nanocrystallization of BRI is a useful tool that can be expected to improve the corneal permeability of BRI. In addition, surfactants have been reported to enhance drug solubility and corneal permeability [15–17], and the commercial formulation, Azopt®, contains tyloxapol as a surfactant. Tyloxapol is a non-ionic surfactant, which is relatively harmless to the eye compared to ionic surfactants [18, 19]. In addition, tyloxapol has been reported to function as a wetting agent and solubilizer, which improves drug solubility and retention on the ocular surface [15–17]. For this reason, tyloxapol is used in several commercial ophthalmic products and has proven to be safe for use [20]. In this study, we investigated the possibility of combining a nanocrystalline formulation of BRI with tyloxapol to achieve synergistic improvement of corneal permeability and enhancement of IOP-reducing effect.

## Methods

### Animals

Seven-week-old male Wistar rats and adult male Japanese White rabbits (weight: 2–3 kg) were purchased from Shimizu Laboratory Supplies Co., Ltd. (Kyoto, Japan). The study was also conducted following the ARRIVE guidelines. The animals were housed under standard conditions (7:00 a.m. to 7:00 p.m. under fluorescent light, 7:00 p.m. to 7:00 a.m. in the dark, at 25 °C). Water, CE-2 (rat), and CE-3 (rabbit) standard diets (Clare Japan, Tokyo, Japan) were freely provided. Animal experiments were conducted following the Pharmacy Committee Guidelines for the Care and Use of Laboratory Animals in the Japanese Pharmacological Society and Kindai University (project identification code No. KASP-2021-003 and KASP-2021-004, approved on April 1, 2021).

### Chemicals

BRI powder was purchased from Nacalai Tesque (Kyoto, Japan). Mannitol (D-mannitol), tyloxapol, methyl *p*-hydroxybenzoate, and isoflurane were purchased from Wako Pure Chemical Industries, Ltd. (Osaka, Japan). Methylcellulose (MC, type SM-4) with an average viscosity of approximately 4 mPa·s at 20 °C was obtained from Shin-Etsu Chemical Co., Ltd. (Tokyo, Japan). Benzalkonium chloride (BAC) was purchased from Kanto Chemical Co., Inc. (Tokyo, Japan) and 2-Hydroxypropyl-β-cyclodextrin (HPβCD) was from Nihon Shokuhin Kako Co., Ltd. (Tokyo, Japan). Azopt® (CA-BRI) was obtained from Novartis Pharma K.K. (Tokyo, Japan) and Benoxil® ophthalmic solution (oxybuprocaine hydrochloride) was from Santen Pharmaceutical Co., Ltd. (Osaka, Japan). All other chemicals used were commercially available in special grade or HPLC grade.

### Preparation of BRI nanocrystal formulations

BRI microcrystal formulation (BRI-MPs) was prepared by mixing 1% BRI powder, 0.8% MC (dispersant), 0.5% D-mannitol (stabilizer), and 0.001% BAC (preservative) in 5.0% HPβCD (solubilizer) aqueous solution, and BRI nanocrystal formulation (BRI-NPs) was prepared by bead-milling the pre-prepared BRI-MPs. The bead-milling was performed under the following conditions using zirconia beads (diameter: 0.1 mm) and Bead Mill NP-100 (Thinky Co., Ltd., Tokyo, Japan): 2,000 rpm for 180 s × 8 times at -20 °C, followed by 400 rpm for 60 s at -20 °C. After milling, we collected the desired suspension, taking care not to take precipitated zirconia beads. The BRI nanocrystal formulation with tyloxapol (Tyl@BRI-NPs) was prepared by adding 0.0005% tyloxapol to BRI-NPs and the BRI microcrystal formulation with tyloxapol (Tyl@BRI-MPs) was also provided using the same protocol.

### Particle size and morphology

The particle size of BRI ophthalmic formulations was measured with the laser diffraction particle size analyzer SALD-7100 (Shimadzu Corp., Kyoto, Japan) and NANOSIGHT LM10 (Malvern, Worcestershire, UK) utilizing nano tracking analysis. The refractive index was set at 1.95 ± 0.05*i* in SALD-7100, and the conditions for NANOSIGHT LM10 were as follows: wavelength, 405 nm; measurement time, 60 s; viscosity, 1.27 mPa∙s. The particle morphology was observed using the scanning probe microscope SPM-9700 (Shimadzu Corp., Kyoto, Japan) and a scanning electron microscope (SEM, NeoScope JCM-7000, JEOL Ltd., Tokyo, Japan).

### Powder X-ray diffractometry (PXRD)

PXRD was performed using MiniFlex 600 (Rigaku Corp., Tokyo, Japan) as the X-ray generator for Cu Kα radiation was used. The samples were prepared by filtering the suspension and drying the filter residue under reduced pressure at room temperature. The PXRD data were collected in the continuous one-dimensional scanning mode with a step size of 0.02 degrees. The scanning speed was 3 degrees/min and the scanned range was 5 to 50 degrees.

### Thermogravimetry and Differential thermal analysis (TG-DTA)

TG-DTA was performed using DTG-60 H (Shimadzu Corp., Kyoto, Japan), under a nitrogen-rich atmosphere (flow rate: 50 mL/min) in aluminum pans. The samples were prepared by filtering the suspension and drying the filter residue under reduced pressure at room temperature. Prepared 5 mg samples were heated from room temperature to 200 °C at a heating rate of 10 °C/min.

### Determination of BRI concentration by HPLC

The concentrations of BRI were determined using an LC-10AD system (HPLC, Shimadzu Corp., Kyoto, Japan) using 0.1 µg/mL methyl *p*-hydroxybenzoate as an internal standard. Inertsil ODS-3 (2.1 mm × 50 mm, GL Science Co., Inc., Tokyo, Japan) was used and the column temperature was 35 °C. The mobile phase consisted of 0.1 M potassium phosphate and acetonitrile (85/15) pumped at a flow rate of 0.25 mL/min for 16.5 min. The detection wavelength was 250 nm. When measuring under the above conditions, the retention time for internal standard and BRI are 3–4 min and 7.5–8.5 min, respectively. The limit of detection (LOD) and quantification (LOQ) of BRI is 0.004 µg/mL and 0.5 µg/mL, respectively.

### Viscosity

Viscosity was measured using a tuning fork vibration viscometer, SV-1 A (A&D Co., Ltd., Tokyo, Japan) at 26 °C. Each formulation was put into a 2 mL tube and the oscillator drive frequency was set to 30 Hz for the measurements.

### Zeta potential

The zeta potential was measured using a micro-electrophoresis zeta potential analyzer (Sanyo Trading Co., Ltd., Tokyo, Japan). Diluted each formulation 20 times with water and measured under the following conditions: electrophoresis distance, 120 μm; strength of electric field, 10 V/cm; sample temperature, 26 °C.

### Dispersal stability for BRI ophthalmic formulations

The experiment was performed according to our previous report [7]. Two milliliters of samples were placed in 3 mL test tubes and incubated in the dark at 20 °C for 28 days. At each sampling point (days 0, 1, 3, 7, 14, and 28), digital images were taken and, after 28 days, the particle size was measured by NANOSIGHT LM10.

### In vitro transcorneal penetration study by using rat cornea

The rats were sacrificed by injecting a lethal dose of pentobarbital (200 mg/kg), and their corneas were removed and placed on a Franz diffusion cell (12.2 mL, Osaka-riko Co., Ltd., Osaka, Japan). The reservoir chamber of the cell was filled with 10 mM HEPES buffer (pH 7.4) containing 136.2 mM NaCl, 5.3 mM KCl, 1.0 mM K_2_HPO_4_, 1.7 mM CaCl_2_, and 5.5 mM glucose, and 20 µL of each BRI ophthalmic formulation was applied into the top of the cell. The experiments were performed at 37 °C. At predetermined times (0–60 min), 200 µL of the HEPES buffer at the reservoir chamber was sampled and immediately replenished with the same volume of buffer in the reservoir chamber. The concentration of BRI in the collected samples was measured under the HPLC conditions described above, and the area under the drug concentration-time curve (*AUC*_0-60 min_) was calculated.

### In vivo transcorneal penetration study

The in vivo transcorneal penetration study of the 1% BRI ophthalmic formulations was determined following our previous reports [7]. Rabbits were anesthetized with isoflurane (flow rate 1.0 L/min, set concentration 3%), and an injection needle (26 G) with a silicon tube (diameter: 0.5 mm) was inserted just below the cornea. After 50 µL of BRI ophthalmic formulations was applied, 5 µL of aqueous humor was collected at predetermined times (0–90 min) and the concentration of BRI in the aqueous humor was measured under the HPLC conditions described above to determine the area under the drug concentration-time curve in the aqueous humor (*AUC*_0-90 min_) according to the trapezoidal rule up to the last BRI concentration measurement point.

### Assessment of corneal damage

Fifty microliters of BRI ophthalmic formulations were instilled in rabbits twice a day (10:00 and 18:00) for one week. Afterward, the rabbit corneas were stained with a solution containing 1% fluorescein and 0.4% oxybuprocaine hydrochloride, and the corneal surface was observed by using a slit lamp METORI-50 V (Seed Corporation, Saitama, Japan) with a blue filter.

### Evaluation of IOP

Ocular hypertension in rabbits was induced as a rapid intravenous injection of 15 mL/kg glucose solution. After 50 µL of 1% BRI ophthalmic formulations were applied, the IOP of rabbits was measured at predetermined times (0–90 min) using an electronic tonometer (Medtronic SOLAN, Jacksonville, FL, USA). IOP was measured thrice repeatedly at a one-time point, and the average IOP at each time point was calculated. *Δ*IOP was calculated from the difference between normal IOP and IOP after the administration of BRI ophthalmic formulations, and the area under the curve of *Δ*IOP versus time (*AUC*_*Δ*IOP_) was calculated according to the trapezoidal rule up to the last IOP measurement point.

### Statistical analysis

The particle size data from SALD-7100 are presented as the mean ± standard deviation, and other data are presented as the mean ± standard error. In this study, Student’s *t*-test and Dunnett’s multiple comparison were used to determine significant differences, and a *P* value less than 0.05 was considered significant.

## Results

### Changes in characteristics of ophthalmic formulation based on the BRI nanocrystals

**Fig. 1:**
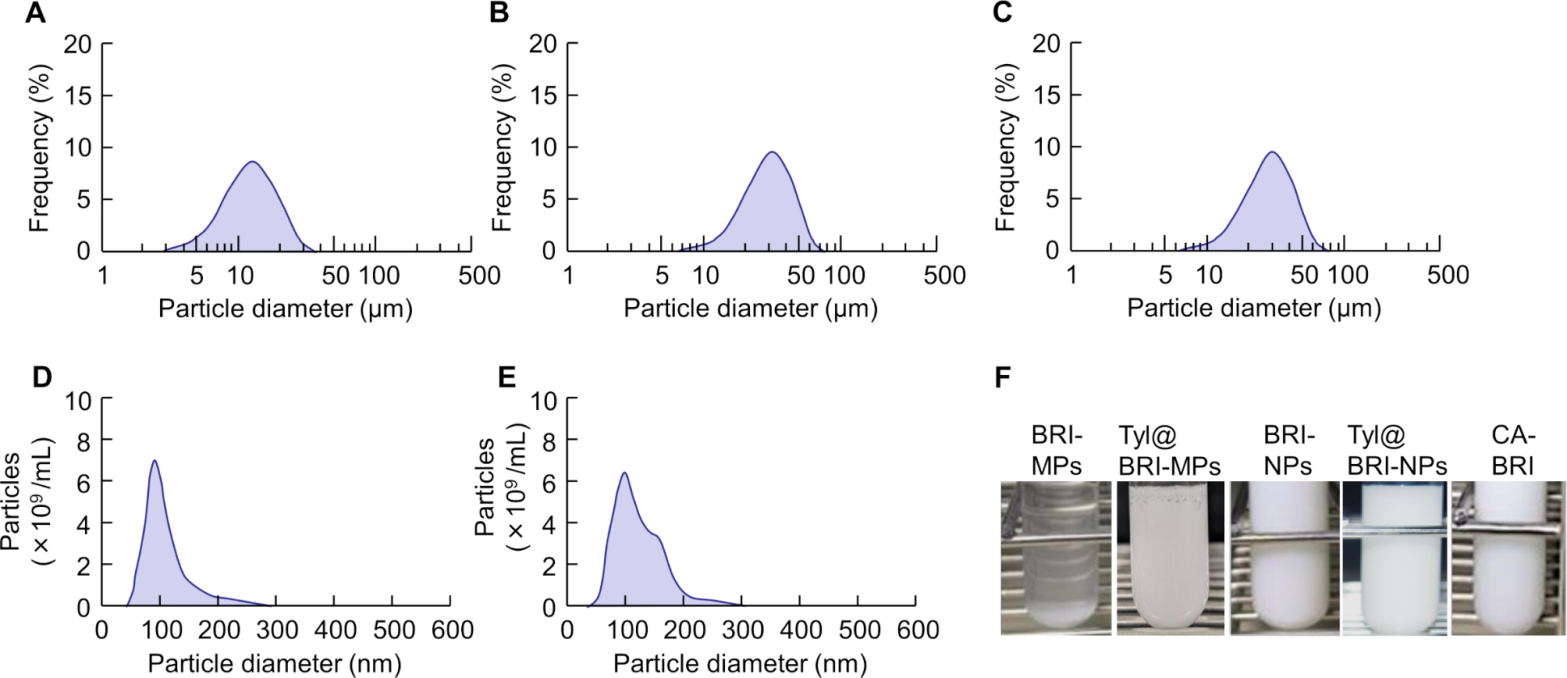
Particle size distribution of CA-BRI (**A**), BRI-MPs (**B**), Tyl@BRI-NPs (**C**), BRI-NPs (**D**), and Tyl@BRI-NPs (**E**) and their representative image (**F**). Figures A - C were measured using SALD-7100, and the particle size distributions of BRI nanoparticles (Fig. D and E) were provided by NANOSIGHT LM10. The mean particle size of each formulation is 10.1 ± 0.318, 27.3 ± 0.250, 24.7 ± 0.241, 0.142 ± 0.003, and 0.135 ± 0.004 μm, respectively

**Fig. 2:**
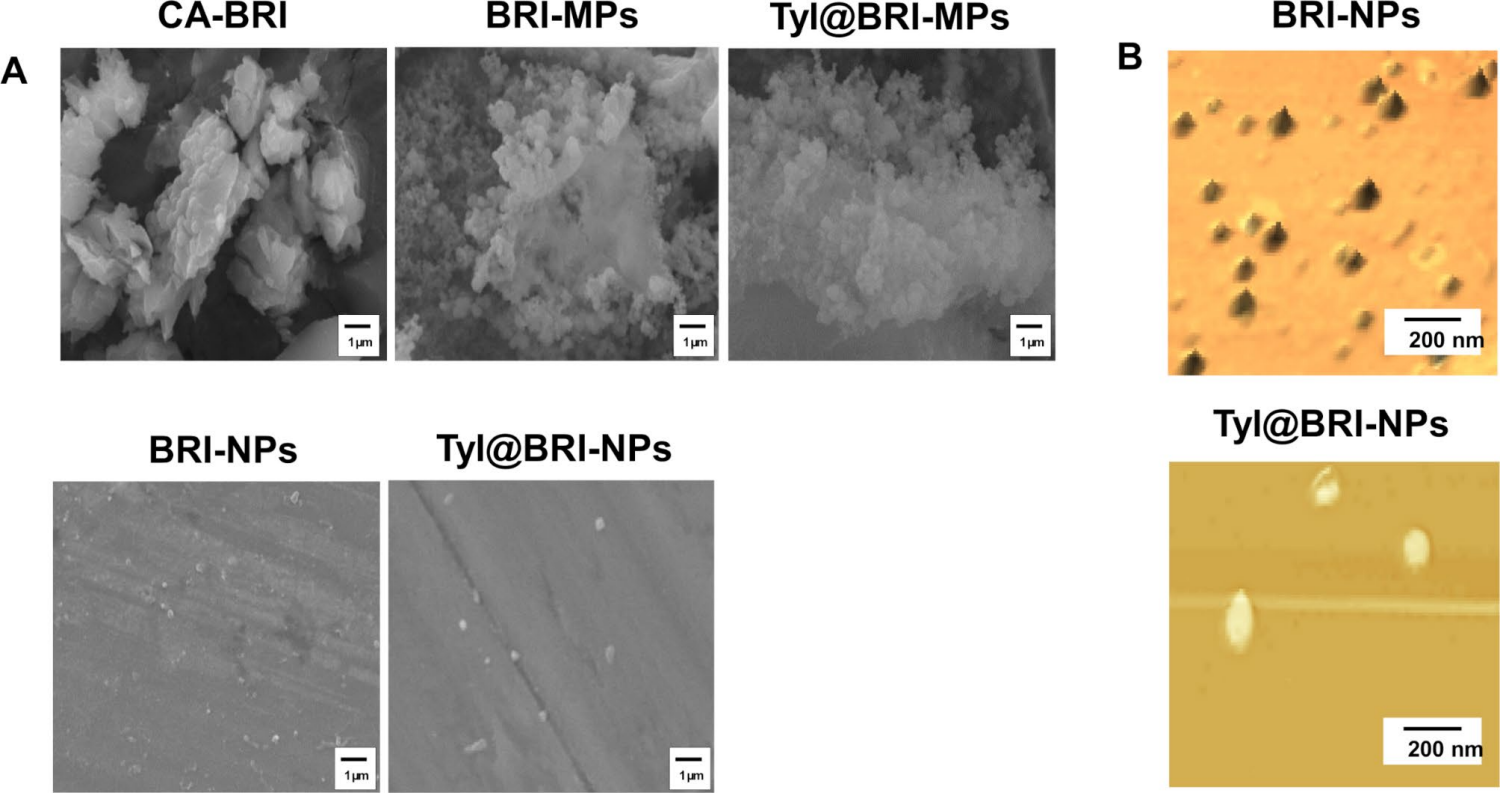
SEM (**A**) and SPM (**B**) images of various BRI ophthalmic formulations taken with JCM-7000 and SPM-9700

**Fig. 3:**
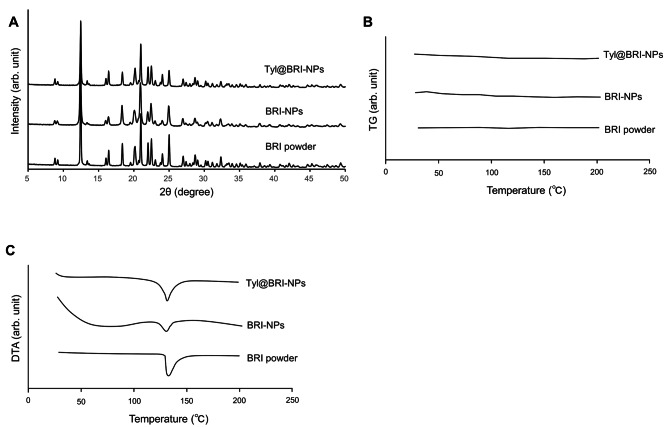
Analysis of the crystal structure of BRI in BRI ophthalmic formulations. (**A**) The PXRD patterns of the commercially available BRI powder, BRI-NPs, and Tyl@BRI-NPs. The reflection intensity scale was set at 0–40,000 cps. (**B**) and (**C**) The TG (**B**) and DTA (**C**) curves of commercially available BRI powder, BRI-NPs, and Tyl@BRI-NPs. The scales of the vertical axis for TG and DTA were set to 0–5 mg and − 25 − 0 mV, respectively

BRI-NPs and Tyl@BRI-NPs were successfully prepared by the wet-milling method with beads and additives. Figure 1 shows the particle size distribution of BRI ophthalmic formulations measured by SALD-7100 and NANOSIGHT LM10. The mean particle sizes of CA-BRI, BRI-MPs, Tyl@BRI-MPs, BRI-NPs, and Tyl@BRI-NPs were 10.1 ± 0.318, 27.3 ± 0.250, 24.7 ± 0.241, 0.142 ± 0.003, and 0.135 ± 0.004 μm, respectively, and the SPAN factor of each formulation were 1.28, 1.13, 1.19 and 1.23, respectively. SPM-9700 and SEM were used to observe the particle morphology. From the results of the SPAN factor and the particle morphology, we confirmed that the nanoparticles of BRI-NPs and Tyl@BRI-NPs were uniformly dispersed (Fig. 2). The results of the PXRD and TG-DTA analyses showed no change in the crystal form of BRI before and after the preparation of BRI-NPs and Tyl@BRI-NPs (Fig. 3). The DTA curves of BRI powder, BRI-NPs and Tyl@BRI-NPs showed only the endothermic peak associated with the melting of BRI (melting point: 131 °C) [13]. Table 1 shows the physical properties of BRI ophthalmic formulations. Generally, nanoparticles improve compound solubility [21, 22], so BRI-NPs and Tyl@BRI-NPs exhibited higher solubility than CA-BRI and BRI-MPs with or without tyloxapol. Moreover, the addition of the surfactant tyloxapol increased the solubility of BRI, and the enhanced solubility was higher for nanocrystals than for microcrystals. Furthermore, BRI-MPs likely exhibited higher solubility than CA-BRI despite the similar particle sizes due to the addition of HPβCD, which enhanced the solubility by forming inclusion complexes [23, 24]. The zeta potential of CA-BRI, BRI-MPs, Tyl@BRI-MPs, BRI-NPs, and Tyl@BRI-NPs were − 61.3, -78.0, -61.5, -69.0, and − 55.3 mV, respectively, and their viscosities were 42.5, 1.66, 1.66, 3.02, and 2.79 mPa∙s, respectively. The viscosity of CA-BRI was significantly higher than those of the others, possibly due to the carboxy vinyl polymer, which is a viscosity agent.

**Table 1:** Physical properties of BRI ophthalmic formulations

Formulations	Solubility (mg/mL)	Zeta potential (mV)	Viscosity (mPa∙s)	Components
BRI-MPs	2.0 ± 0.2	−78.0 ± 2.1	1.66 ± 0.1	1% BRI, 0.8% Methyl cellulose, 0.5% D-mannitol, 0.001% BAC, 5% HPβCD
Tyl@BRI-MPs	2.5 ± 0.0	−61.5 ± 1.5	1.66 ± 0.1	1% BRI, 0.8% Methyl cellulose, 0.5% D-mannitol, 0.001% BAC, 5% HPβCD, 0.0005% Tyloxapol
BRI-NPs	3.0 ± 0.2	−69.0 ± 2.7	3.02 ± 0.1	1% BRI, 0.8% Methyl cellulose, 0.5% D-mannitol, 0.001% BAC, 5% HPβCD
Tyl@BRI-NPs	4.4 ± 0.1	−55.3 ± 1.0	2.79 ± 0.1	1% BRI, 0.8% Methyl cellulose, 0.5% D-mannitol, 0.001% BAC, 5% HPβCD, 0.0005% Tyloxapol
CA-BRI	0.5 ± 0.0	−61.3 ± 1.2	42.5 ± 0.3	1% BRI, Carboxy vinyl polymer, Tyloxapol, D-mannitol, EDTA, BAC

Mean ± S.E., Solubility *n* = 3, zeta potential *n* = 10, viscosity *n* = 5

### Changes in dispersal stability for BRI ophthalmic formulations

**Fig. 4:**
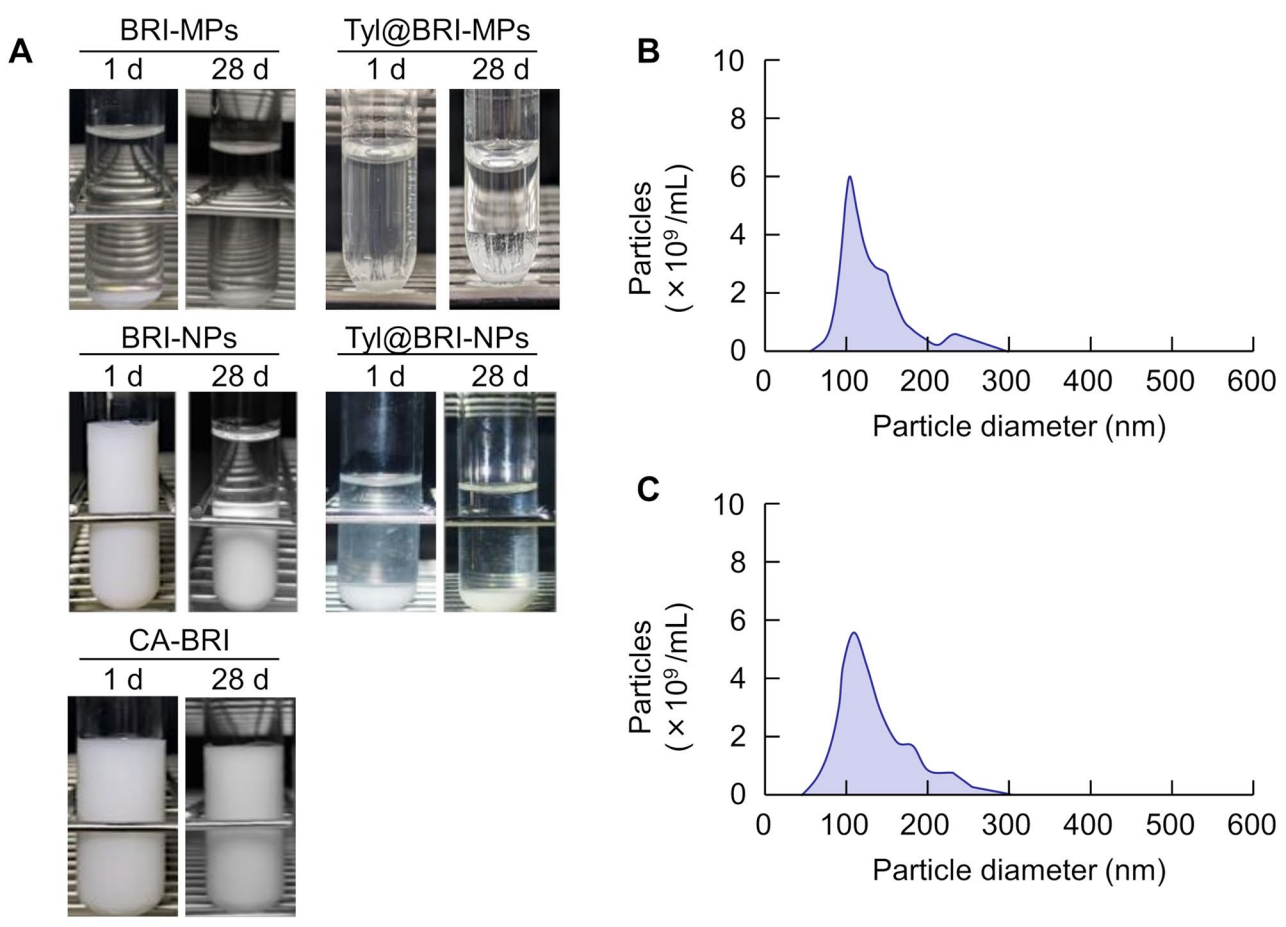
Changes in the dispersibility (**A**) and particle size distribution of BRI-NPs (**B**) and Tyl@BRI-NPs (**C**) after 28 days as measured by NANOSIGHT LM10

The results of dispersal stability for BRI ophthalmic formulations are shown in Fig. 4. BRI-MPs showed poor dispersibility, with particles settling immediately after preparation, but BRI-NPs exhibited improved dispersibility after nanocrystallization and particle settling was suppressed. Although two layers of separation were observed in the BRI-NPs 28 days after preparation, the particles of BRI remained nano-sized even after 28 days. This result suggests that BRI-NPs were highly redispersible. In Tyl@BRI-MPs and Tyl@BRI-NPs, in which tyloxapol was added to BRI, the dispersibility was significantly reduced. However, Tyl@BRI-NPs returned to suspension by stirring, where the drug particles were nano-sized. Meanwhile, no sedimentation of BRI particles was observed in CA-BRI even after 28 days, which may be because its viscosity was 14 times higher than that of BRI-NPs.

### In vitro transcorneal penetration of BRI ophthalmic formulations

**Fig. 5:**
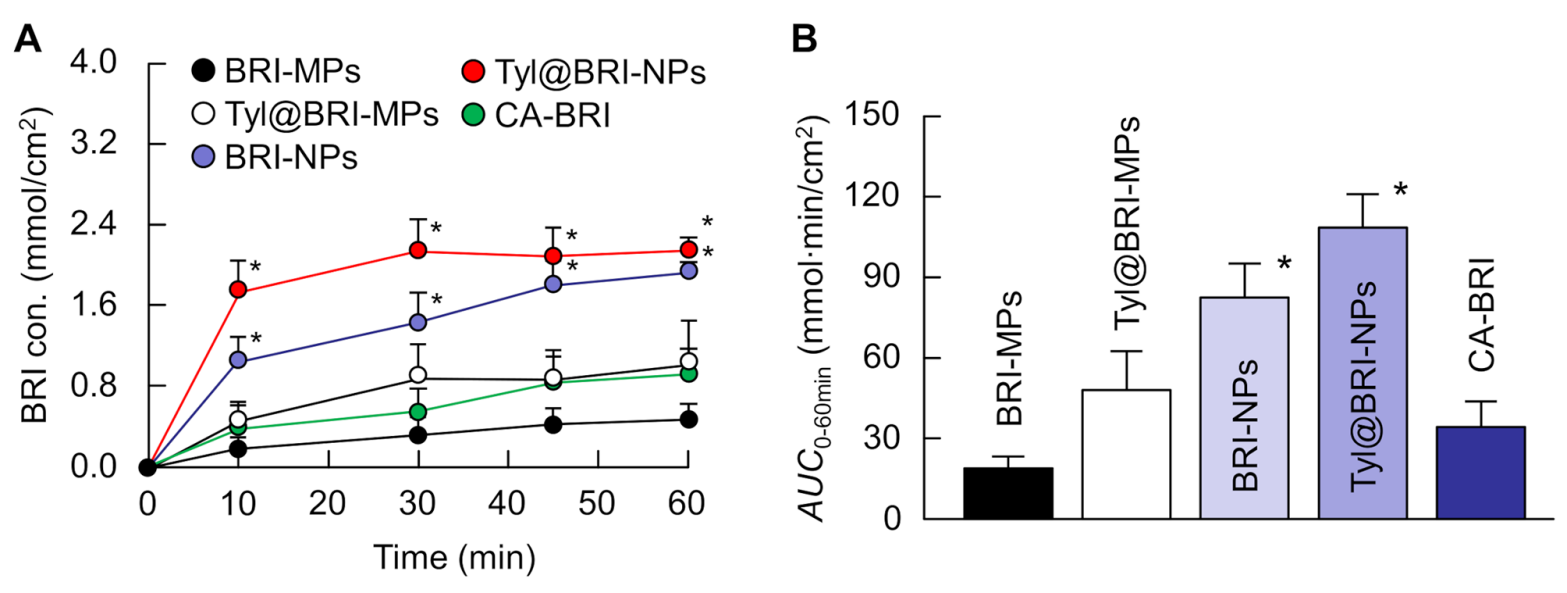
In vitro transcorneal penetration (**A**) and *AUC*_0 − 90 min_ (**B**) of CA-BRI, BRI-MPs, Tyl@BRI-MPs, BRI-NPs, and Tyl@BRI-NPs using the corneas of rats. Mean ± SE, *n* = 5–10. *P < 0.05 vs. CA-BRI

Figure 5 shows the in vitro transcorneal penetration of BRI-MPs, Tyl@BRI-MPs, BRI-NPs, Tyl@BRI-NPs, and CA-BRI using the isolated corneas of rats. The in vitro transcorneal penetration of BRI-MPs, Tyl@BRI-MPs and CA-BRI was similar and BRI-NPs was 4.2- and 2.4-fold those of BRI-MPs and CA-BRI, respectively. Interestingly, the corneal permeability of BRI-NPs was further enhanced by the addition of tyloxapol, and the enhancement of corneal permeability in the combination of BRI nanocrystals and tyloxapol (Tyl@BRI-NPs) was obviously higher than that in BRI microcrystals and tyloxapol (Tyl@BRI-MPs).

### In vivo transcorneal penetration of BRI ophthalmic formulations

**Fig. 6:**
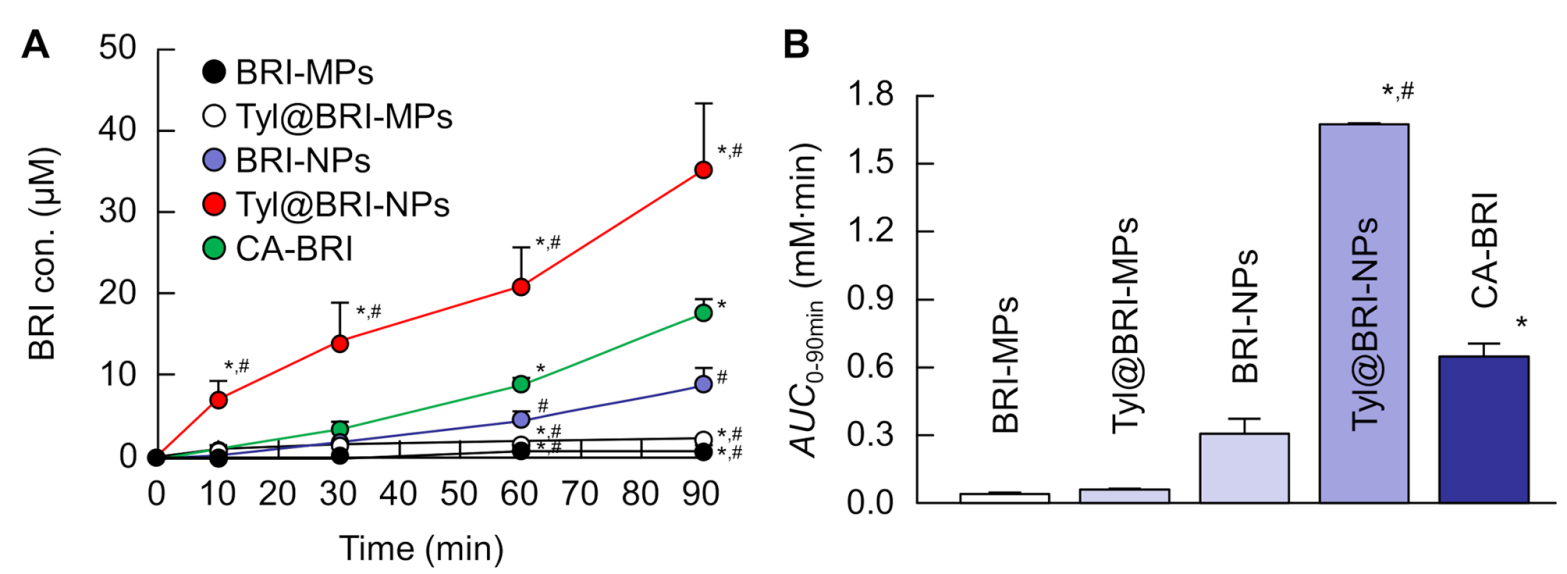
In vivo transcorneal penetration (**A**) and *AUC*_0 − 90 min_ (**B**) of CA-BRI, BRI-MPs, Tyl@BRI-MPs, BRI-NPs, and Tyl@BRI-NPs using rabbits. Mean ± SE, *n* = 5–7. *P < 0.05 vs. BRI-NPs. ^#^*P* < 0.05 vs. CA-BRI

The concentration of BRI in the aqueous humor of rabbits when each formulation was instilled is shown in Fig. 6. Comparing BRI-MPs and BRI-NPs, the *AUC*_0 − 90 min_ calculated from the BRI concentration in the aqueous humor was 7.71 times higher for BRI-NPs than for BRI-MPs, indicating significantly improved corneal permeability due to nanocrystallization. However, CA-BRI, micro-sized particles, showed higher corneal permeability than BRI-NPs, and the addition of 0.0005% tyloxapol to BRI-NPs (Tyl@BRI-NPs) resulted in a 2.60-fold higher *AUC*_0 − 90 min_ than CA-BRI. The *AUC*_0 − 90 min_ of Tyl@BRI-NPs was 28.2-fold higher *AUC*_0 − 90 min_ than Tyl@BRI-MPs.

### Therapeutic effect of BRI ophthalmic nanoformulations on IOP in rabbits

**Fig. 7:**
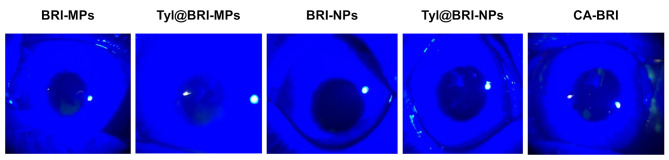
Changes in corneal toxicity in a rabbit instilled with various BRI ophthalmic formulations

**Fig. 8:**
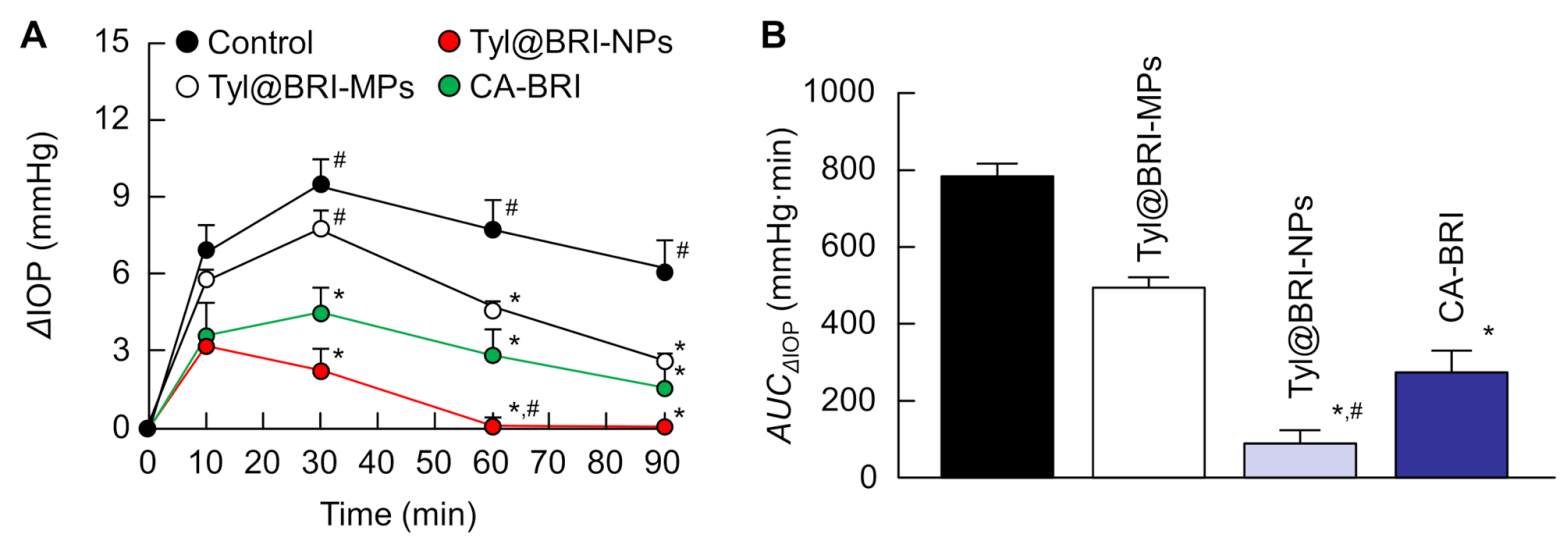
Effect of BRI ophthalmic formulations on the IOP-reducing effect in rabbits with high IOP. (**A**) Changes in ⊿IOP of BRI formulations. (**B**) Changes in *AUC*_⊿IOP_ of BRI formulations. Mean ± SE, *n* = 5. *P < 0.05 vs. Control. ^#^*P* < 0.05 vs. CA-BRI

The BRI ophthalmic nanoformulations must be safe for use in the ocular field. In this study, we investigated whether the repetitive instillation of BRI ophthalmic formulations causes corneal toxicity (Fig. 7). As a result, no corneal damage was observed with repeated instillation of either formulation (BRI-MPs, Tyl@BRI-MPs, BRI-NPs, Tyl@BRI-NPs and CA-BRI). Afterward, we demonstrated the IOP-reducing effect of Tyl@BRI-NPs and CA-BRI. The results of ΔIOP (mmHg) and *AUC*_ΔIOP_ when each formulation was instilled in rabbits with high IOP is shown in Fig. 8. The BRI administered by eye drops reduces IOP by penetrating the cornea and inhibiting carbonic anhydrase in the ciliary body as in previous reports [12]. Based on the results of the in vivo corneal permeability study (Fig. 6), we expected that the order of the IOP-reducing effect would be Tyl@BRI-NPs > CA-BRI > Tyl@BRI-MPs, the former having a higher corneal permeability. As expected, Tyl@BRI-NPs showed the strongest IOP-reducing effect.

## Discussion

In this study, we prepared BRI nanocrystal formulations and investigated the possibility of improving the efficacy of glaucoma treatment by ophthalmic administration. Nanocrystal preparation methods are mainly classified into top-down (nanonization) and bottom-up (crystal growth or nucleation) methods, and the top-down methods are more straightforward to scale up than the bottom-up methods; uniform-sized nanocrystals can be prepared [25]. We have previously reported the nanocrystallization of drugs using the media milling method, one of the top-down methods, and found that a combination of MC, D-mannitol, BAC, and HPβCD was useful as a medium [7, 9]. Therefore, BRI nanocrystallization was performed under similar conditions, and BRI-NPs and Tyl@BRI-NPs were successfully prepared with the mean particle sizes were 0.142 ± 0.003, and 0.135 ± 0.004 μm, respectively (Fig. 1). Although it is known that differences in the crystal form of drugs affect their solubility and stability [26], the results of the XRPD and DTA analyses showed no change in the crystal form of BRI before and after the preparation of BRI-NPs and Tyl@BRI-NPs (Fig. 3).

The corneal permeability of the prepared nanocrystal formulations were evaluated by both in vitro system in which rat corneas were placed on a Franz diffusion cell and in vivo system which was instilled in rabbits by eye drops. In in vivo corneal permeability study, it is not possible to evaluate whether the improved intraocular migration of BRI is due to improved retention on the ocular surface or to improved corneal permeability. In in vitro corneal permeability study, on the other hand, BRI is retained on the corneal surface and only the effect of corneal permeability can be evaluated. From the results of the in vitro study, BRI-NPs showed higher BRI concentration in the reservoir chamber than CA-BRI and BRI-MPs, and in the in vivo study, the concentration of BRI in the aqueous humor after eye drops was higher for BRI-NPs than for BRI-MPs. These results suggest that nanocrystallization significantly improves the corneal permeability of BRI. The improvement of BA by nanocrystallization not only improves drug solubility but also involves energy-dependent endocytosis induced by the attachment of nanoparticles to cell membranes [27, 28]. The uptake of nanoparticles into cells by endocytosis primarily involves caveolae-dependent endocytosis (CavME), clathrin-dependent endocytosis (CME), and macropinocytosis (MP), which facilitate the uptake of particles less than 80 nm, less than 120 nm, and 100 nm to 5 μm in size, respectively [29, 30]. We have previously shown that nanocrystal formulations of Indomethacin exhibit high corneal permeability via the endocytosis mechanism [31], and BRI-NPs likely also exhibit high corneal permeability via the same mechanism.

Meanwhile, CA-BRI exhibited higer corneal permeability than BRI-NPs in the in vivo study. In generally, increasing the viscosity of the formulation is known to improve precorneal residence time and hence a greater transcorneal penetration of the drug [32]. Toropaonen et al. reported that increasing the viscosity of indomethacin ophthalmic suspension from 1.3 to 15 mPa resulted in about 4-fold increase in intraocular migration [33]. The viscosity and dispersibility results (Table 1; Fig. 4) suggest that the drug retention of CA-BRI on the ocular surface is better than that of other formulations, and the fact that CA-BRI is less susceptible to lacrimal fluid clearance may be one of the reasons for its high corneal permeability. On the other hand, in contrast to in vivo results, the corneal permeability of CA-BRI in the in vitro study was low. This may be because the in vitro system is not affected by tear fluid clearance and the precorneal residual time of BRI is the same regardless of formulation characteristics such as viscosity and dispersion.

To further improve the corneal permeability of BRI-NPs, we prepared Tyl@BRI-NPs, in which 0.0005% tyloxapol was added to BRI-NPs. Tyloxapol is described as a non-ionic liquid surfactant of the alkyl-aryl-polyether alcohol type in the official monographs of the USP (USP43-NF38, 4551), and is also contained in CA-BRI. Tyloxapol is a non-ionic molecule composed of both polar and non-polar segments, that functions as a wetting agent and solubilizer, which is responsible for improving drug solubility and retention on the ocular surface [15–17]. It has also been suggested that surfactants can be incorporated into the phospholipid bilayer of corneal epithelial cells and form mixed micelles with phospholipids, thereby inducing solubilization of the cell membrane and improving drug permeability [15]. In additon, non-ionic surfactants are relatively harmless to the eye compared to ionic surfactants when used properly [18, 19]. Tyloxapol is also used as an additive in several commercial ophthalmic products and has proven to be safe for use [20]. Therefore, we performed the corneal permeability study of Tyl@BRI-NPs, it greatly enhanced the corneal permeability of BRI in the in vivo study, resulting in the *AUC*_0 − 90 min_ was 2.60 times higher than in CA-BRI (Fig. 6). In addition, the enhancement of corneal permeability in the combination of BRI nanocrystals and tyloxapol (Tyl@BRI-NPs) was obviously higher than that in BRI microcrystals and tyloxapol (Tyl@BRI-MPs). This result suggests that the combination of drug nanocrystals and tyloxapol synergistically improves corneal permeability. On the other hand, it is assumed that nanocrystallization and tyloxapol addition increase the risk of systemic exposure of BRI. In previous studies, it has been reported that oral administration of 2 mg of BRI per day did not cause systemic side effects [12], and the daily dose of BRI in this study is 1 mg, so there is no risk of systemic side effects with ophthalmic administration of Tyl@BRI-NPs.

The BRI administered by eye drops reduces IOP by penetrating the cornea and inhibiting carbonic anhydrase in the ciliary body as in previous reports [12]. Therefore, we demonstrated the IOP-reducing effect of Tyl@BRI-NPs, which had the highest drug delivery into the aqueous humor in the in vivo transcorneal penetration study. The results showed that ophthalmic administration of Tyl@BRI-NPs significantly reduced IOP, and was more effective than Tyl@BRI-MPs and CA-BRI. In addition, no corneal damage was observed during the repetitive instillation of Tyl@BRI-NPs. Based on these results, Tyl@BRI-NPs can be expected to have stronger glaucoma therapeutic effects than CA-BRI without increasing corneal damage. On the other hand, further studies are required to assure the sterility of the formulation for the practical application of BRI nanocrystal formulations in the future.

## Conclusion

We successfully prepared BRI nanocrystal formulations by the media milling method, and the results of the in vivo corneal permeation study showed that Tyl@BRI-NPs, a BRI nanocrystal formulation with tyloxapol, had higher corneal permeability than the commercial drug. In addition, Tyl@BRI-NPs reduced IOP in the ocular hypertension rabbit more than CA-BRI and is expected to have strong glaucoma therapeutic effects. In conclusion, the results of this study demonstrate that the combination of BRI nanocrystallization and tyloxapol is expected to be highly effective in glaucoma treatment and a useful tool for new ophthalmic drug delivery.

## Data Availability

The data generated in the present study may be requested from the corresponding author.

## Abbreviations

BA: Bioavailability
BAC: Benzalkonium chloride
BRI: Brinzolamide
BRI-MPs: BRI microcrystal formulation
BRI-NPs: BRI nanocrystal formulation
CAI: carbonic anhydrase inhibitor
CavME: Caveolae-dependent endocytosis
CME: Clathrin-dependent endocytosis
DDS: Drug delivery systems
DTA: Differential thermal analysis
HPβCD: 2-Hydroxypropyl-β-cyclodextrin
MC: Methylcellulose
IOP: Intraocular pressure
MP: Micropinocytosis
POAG: Primary open-angle glaucoma
SEM: Scanning electron microscope
Tyl@BRI-NPs: BRI nanocrystals and tyloxapol based formulation
XRPD: X-ray powder diffractometry
